# Nesprins: Tissue-Specific Expression of Epsilon and Other Short Isoforms

**DOI:** 10.1371/journal.pone.0094380

**Published:** 2014-04-09

**Authors:** Nguyen Thuy Duong, Glenn E. Morris, Le Thanh Lam, Qiuping Zhang, Caroline A. Sewry, Catherine M. Shanahan, Ian Holt

**Affiliations:** 1 Wolfson Centre for Inherited Neuromuscular Disease, RJAH Orthopaedic Hospital, Oswestry, United Kingdom; 2 Institute of Genome Research (IGR), Vietnam Academy of Science and Technology (VAST), Hanoi, Vietnam; 3 Institute for Science and Technology in Medicine, Keele University, Staffordshire, United Kingdom; 4 Cardiovascular Division, James Black Centre, King’s College, London, United Kingdom; 5 Dubowitz Neuromuscular Centre, Institute for Child Health and Great Ormond Street Hospital, London, United Kingdom; University of Vienna, Max F. Perutz Laboratories, Austria

## Abstract

Nesprin-1-giant and nesprin-2-giant regulate nuclear positioning by the interaction of their C-terminal KASH domains with nuclear membrane SUN proteins and their N-terminal calponin-homology domains with cytoskeletal actin. A number of short isoforms lacking the actin-binding domains are produced by internal promotion. We have evaluated the significance of these shorter isoforms using quantitative RT-PCR and western blotting with site-specific monoclonal antibodies. Within a complete map of nesprin isoforms, we describe two novel nesprin-2 epsilon isoforms for the first time. Epsilon isoforms are similar in size and structure to nesprin-1-alpha. Expression of nesprin isoforms was highly tissue-dependent. Nesprin-2-epsilon-1 was found in early embryonic cells, while nesprin-2-epsilon-2 was present in heart and other adult tissues, but not skeletal muscle. Some cell lines lack shorter isoforms and express only one of the two nesprin genes, suggesting that either of the giant nesprins is sufficient for basic cell functions. For the first time, localisation of endogenous nesprin away from the nuclear membrane was shown in cells where removal of the KASH domain by alternative splicing occurs. By distinguishing between degradation products and true isoforms on western blots, it was found that previously-described beta and gamma isoforms are expressed either at only low levels or with a limited tissue distribution. Two of the shortest alpha isoforms, nesprin-1-alpha-2 and nesprin-2-alpha-1, were found almost exclusively in cardiac and skeletal muscle and a highly conserved and alternatively-spliced exon, available in both nesprin genes, was always included in these tissues. These “muscle-specific” isoforms are thought to form a complex with emerin and lamin A/C at the inner nuclear membrane and mutations in all three proteins cause Emery-Dreifuss muscular dystrophy and/or inherited dilated cardiomyopathy, disorders in which only skeletal muscle and/or heart are affected.

## Introduction

Nesprins (nuclear envelope spectrin-repeat proteins) are intracellular linkers and scaffolds. The *SYNE1* gene for nesprin-1 was first identified in the mouse post-synaptic membrane [1] and in rat vascular smooth muscle cells [2]. Two protein products were postulated, one of approximately 110 kD and another greater than 230 kD [1], [2]. Zhang et al., 2001 [2] named the equivalent human 110 kD protein, nesprin-1-alpha, and identified the larger product as 382 kD nesprin-1-beta. A related gene, *SYNE2*, was also identified [1], [2], and shown to produce protein products of approximately 61 kD (nesprin-2-alpha), 87 kD (nesprin-2-beta) and 377 kD (nesprin-2-gamma) [2]. It was shown that these nesprins are short forms of larger proteins, nesprin-1-giant (1008 kD) and nesprin-2-giant (792 kD) [2]–[4]. *SYNE1*, also known as *MYNE1* [5] or *Enaptin* [6], is on human chromosome 6q25. *SYNE2*, also known as *NUANCE* [3], is on human chromosome 14q23.

Structurally, nesprins have a central rod domain composed of spectrin repeats. Nesprin-1-giant and nesprin-2-giant have N-terminal CH (calponin homology) domains that bind the actin cytoskeleton and C-terminal transmembrane KASH (Klarsicht-ANC-Syne-homology) domains, which reside in the outer nuclear membrane and bind across the luminal space to the SUN (Sad1, UNC84) type II inner nuclear membrane proteins, SUN1 and SUN2 [7], [8]. These interactions form LINC (Linker of Nucleoskeleton and Cytoskeleton) complexes, which form a physical link between the cytoskeleton and the nucleus (Reviewed: [9], [10]). Nesprins have multiple internal promoters which give rise to shorter isoforms with a common C-terminal region, but truncated at the N-terminus. Those short isoforms containing the transmembrane KASH domain are also able to interact with the SUN proteins. The LINC complex is further strengthened by SUN proteins interacting with lamin A/C on the nucleoplasmic side of the inner nuclear membrane. Additionally, the spectrin repeat-containing domain of nesprin-2-alpha ΔTM, which may be nucleoplasmic, has been shown to interact with a different domain of the SUN proteins, so that SUN may anchor nesprin isoforms on opposing faces of the nuclear envelope [11].

The nesprins can undergo alternative splicing to give rise to proteins lacking the KASH and transmembrane domains. These KASH-less isoforms are unable to form LINC complexes with SUN proteins, but can participate in the direct interactions of nesprins in the nucleoplasm. If the KASH transmembrane domains are absent, there may then be direct interactions between the C-terminal regions of nesprins 1 and 2 and their binding partners including emerin [4], [12], muscle A–kinase anchoring protein (encoded by gene *AKAP6*) [13], components of the nuclear lamina (Lamin A/C) [4], [5], [12] a muscle-specific tyrosine kinase receptor (encoded by gene *MuSK*) [1] and chromatin [2], [4].

The bioinformatics study of Simpson and Roberts [14] found good support for two additional nesprin-1 isoforms, with little known about their function, which are not included in this study because they contain only N-terminal sequences (CPG2∶109 kD and GSRP-56∶56 kD). The two additional members of the nesprin family (nesprin-3 and -4), which are not included in this study, both lack the N-terminal CH domain. Instead, the N-terminal of nesprin-3 contains a plectin-binding domain which interacts with intermediate filaments [15] and nesprin-4 interacts with microtubules via kif5b and is involved in cell polarity [16].

Mutations in the C-terminal regions of nesprins-1 and -2 have been associated with Emery-Dreifuss muscular dystrophy (EDMD) and dilated cardiomyopathy (CMD) [17]–[19]. Similarly, mutations in nesprin-binding partners, emerin (*EMD*) [20] and lamin A/C (*LMNA*) [21] are also associated with EDMD (reviewed: [22]). Mutations in two other genes, four and a half LIM domains 1 (*FHL1*) and transmembrane protein 43 (*TMEM43*), may also cause EDMD [23], [24]. FHL1 proteins contain LIM (Lin-11, Isl-1, Mec3) domains and the main isoform, FHL1A is expressed predominantly in striated muscle where it may have a role in sarcomere assembly [23]. *TMEM43* encodes for LUMA, a structural protein of the inner nuclear membrane that interacts with lamins and emerin [24]. In around half of cases of EDMD, causative mutations have not been identified [22]. The characteristic features of EDMD are weakness and wasting of specific muscles, early contractures and cardiac conduction defects [25], but the molecular mechanisms by which the mutations in emerin, lamins or nesprins lead to the clinical features of EDMD are still largely unknown.

Studies of the short isoforms of nesprin-1 and nesprin-2 have often been inconclusive, because of the possibility that some bands seen on northern and western blots may be the result of degradation of endogenous mRNAs and proteins in tissue extracts, rather than the detection of true short isoforms. In the present study, taking as a starting point the bioinformatics data of Simpson and Roberts [14], we have defined more fully the ‘nesprinome’ of different tissue types. We have re-evaluated the importance of previously-reported short isoforms of nesprin-1 and nesprin-2 that have a common C-terminal domain, by determining their expression levels relative to “housekeeping” proteins and to the giant, full-length nesprin proteins. We show that some short isoforms are expressed at very low, or barely-detectable, levels in most tissues, though, in some cases, they may be significant in certain specific cells or tissues. Examples of how degradation products of giant nesprins may have been mistaken for true isoforms are given. In contrast, we also show that the importance of two novel epsilon isoforms of nesprin-2 has been previously overlooked. Finally, the abundant expression of one specific alpha isoform of each nesprin in both cardiac and skeletal muscles suggests that these may be important for understanding the pathogenesis of EDMD.

## Results

**Figure 1:**
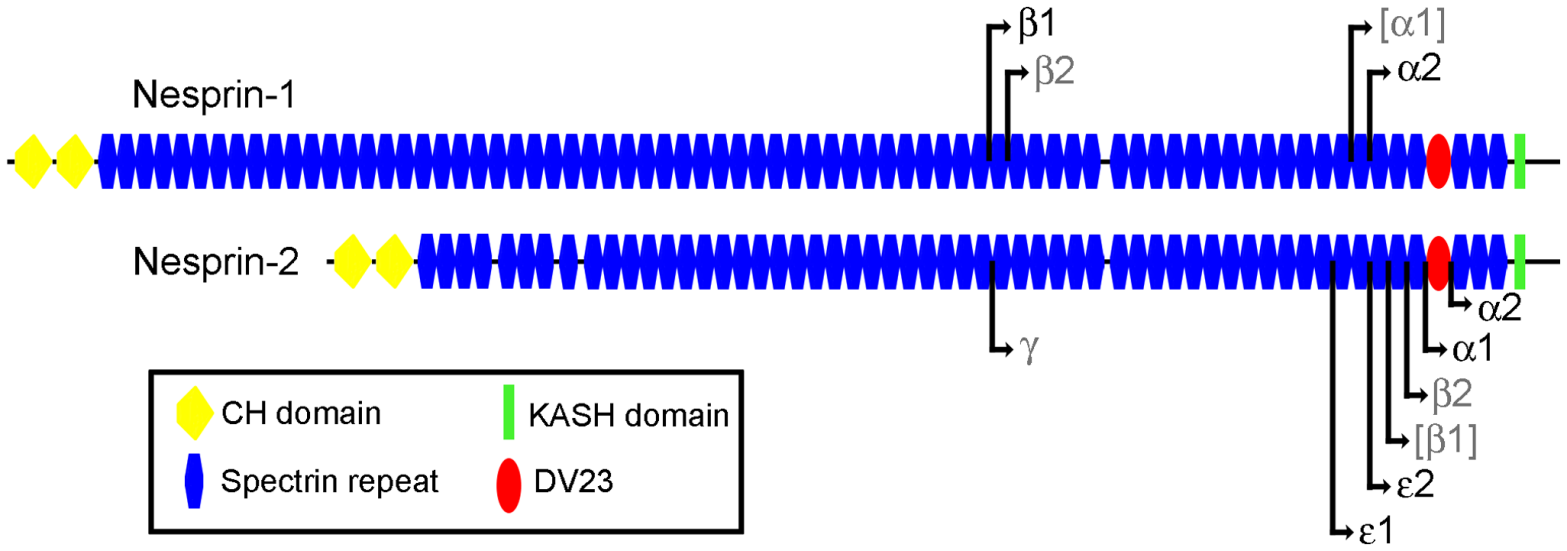
Short forms of nesprin-1 and nesprin-2. Pictorial representation of nesprin-1-giant and nesprin-2-giant with the N-terminal start points of the smaller isoforms indicated by black lines and arrows. Isoforms that we found present at low levels are labelled in grey. Those isoforms that were barely detectable, or undetectable, are labelled in grey with parenthesis. (See Results).

Figure 1 is a pictorial representation of nesprin-1-giant and nesprin-2-giant with the N-terminal start points of the smaller isoforms, as defined by Simpson and Roberts [14]. To determine isoform mRNA levels, we performed qPCR on total cDNA from a panel of 20 human tissues and 7 human cell lines and calculated a Relative Expression (RE) value against two endogenous “house-keeping” controls, GAPDH and cytoskeletal beta-actin. The isoforms, isoform-specific primer sequences, product sizes and efficiencies of amplification are shown in Table S1 in File S1. Primer pair specificity was verified by sequencing the products from conventional PCR. Although a PCR product for sequencing was obtained for nesprin-1-alpha-1 (in spleen) and nesprin-2-beta-1 (in skeletal muscle), we were unable to determine an efficiency of qPCR with any tissue, even when several alternative primer pairs were tested. This suggests that nesprin-1-alpha-1 and nesprin-2-beta-1 were present at very low or undetectable levels in the 27 cells/tissues we have studied. Otherwise, the high PCR efficiencies show that qPCR is accurately reflecting the levels of each mRNA species, relative to the internal controls.

**Figure 2:**
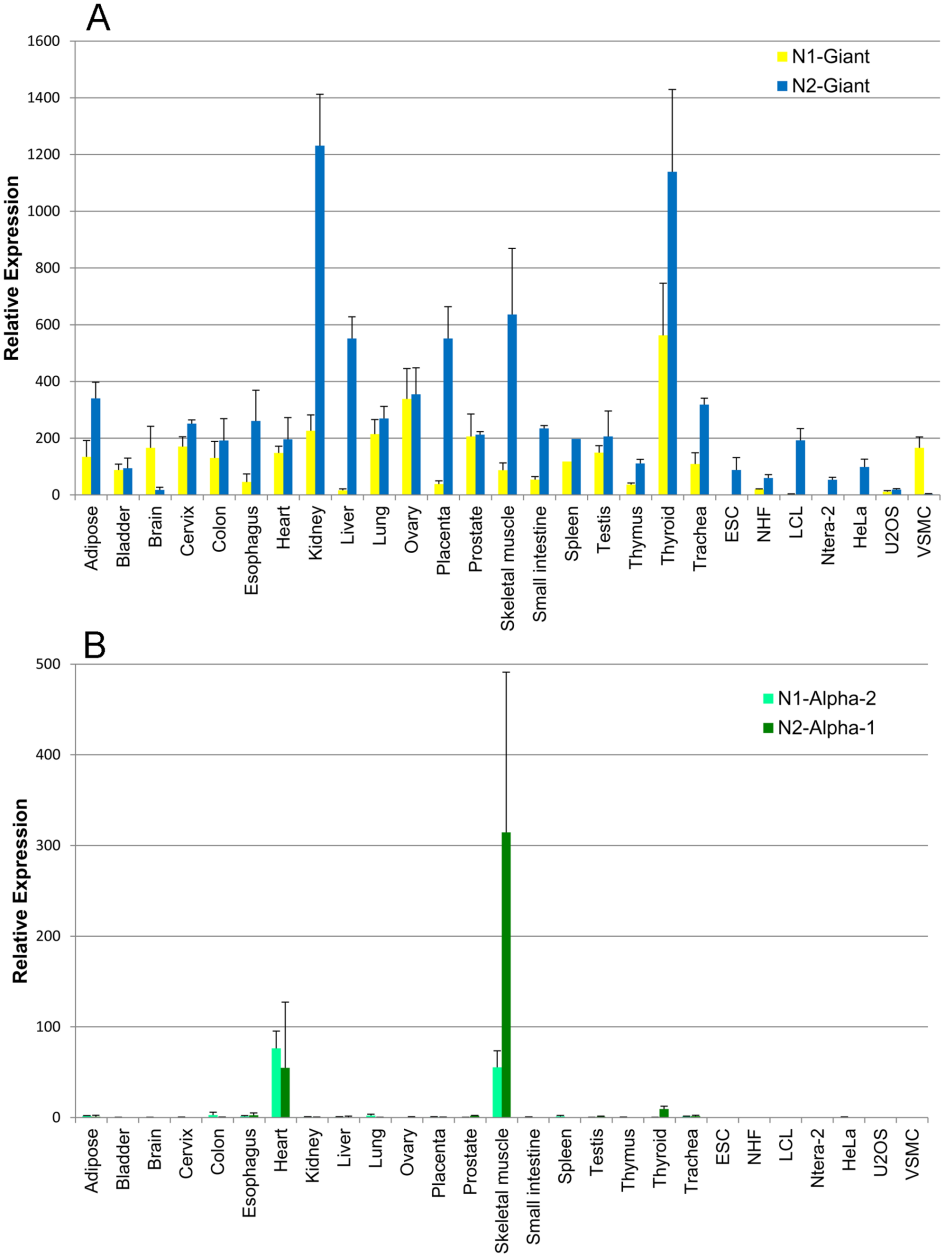
Tissue-specific expression of giant and alpha isoforms of nesprins. Quantitative PCR to show mRNA expression of nesprin isoforms relative to the expression of two endogenous house-keeping controls. Charts represent the mean relative expression ± SEM, measured in cDNA preparations from 20 human tissues and 7 cell lines. These values are given in Tables S3 and S4 in File S1. Bar charts show relative expression of (A) nesprin-1-giant and nesprin-2-giant, and (B) nesprin-1-alpha-2 and nesprin-2-alpha-1.

**Figure 3:**
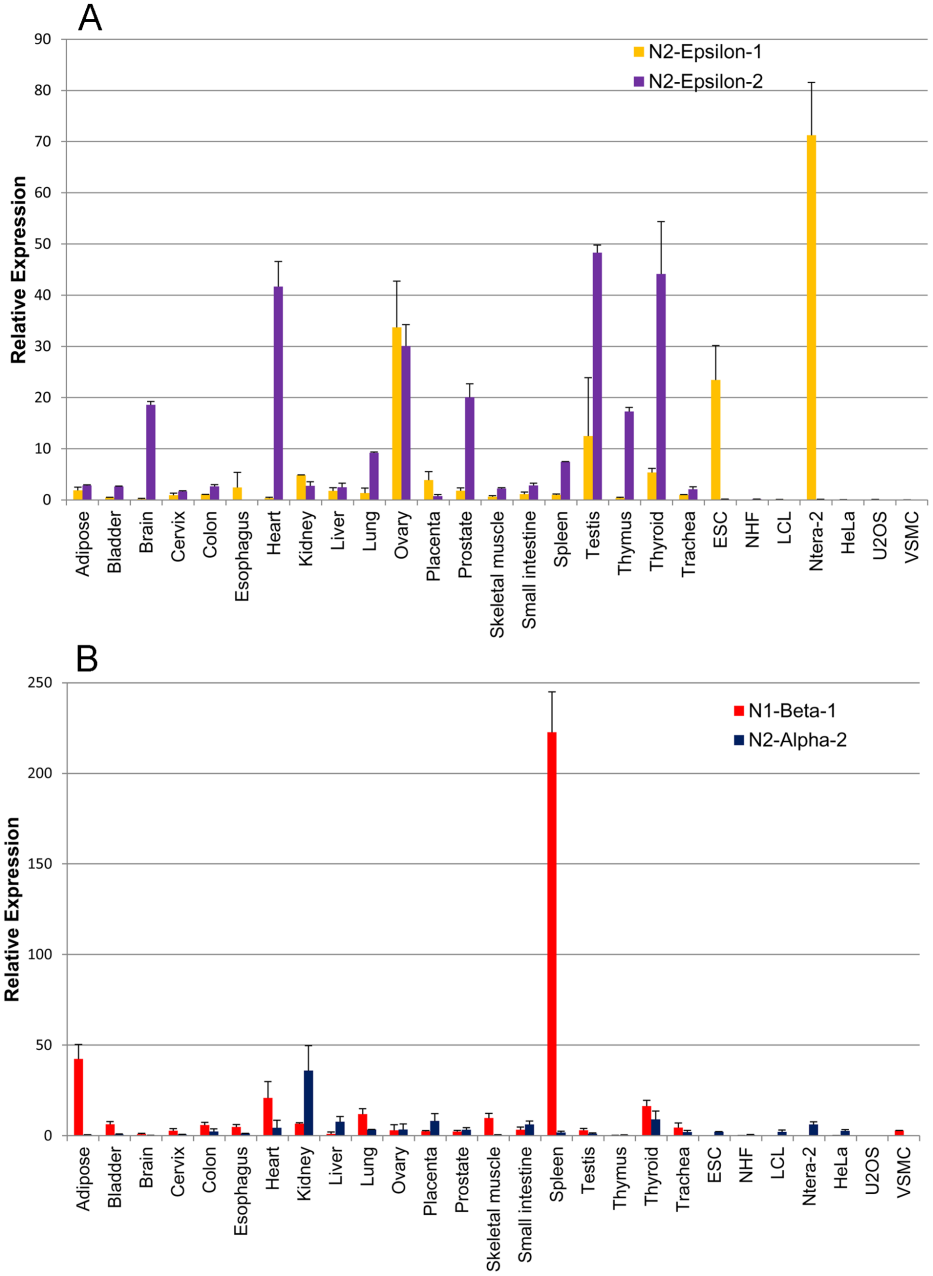
Tissue-specific expression of nesprin-1-beta-1, nesprin-2-alpha-2 and the two nesprin-2-epsilon isoforms. Quantitative PCR to show mRNA expression of nesprin isoforms relative to the expression of two endogenous house-keeping controls. Charts represent the mean relative expression ± SEM, measured in cDNA preparations from 20 human tissues and 7 cell lines. These values are given in Tables S3 and S4 in File S1. Bar charts show relative expression of (A) nesprin-2-epsilon-1 and nesprin-2-epsilon-2, and (B) nesprin-1-beta-1 and nesprin-2-alpha-2.

**Figure 4:**
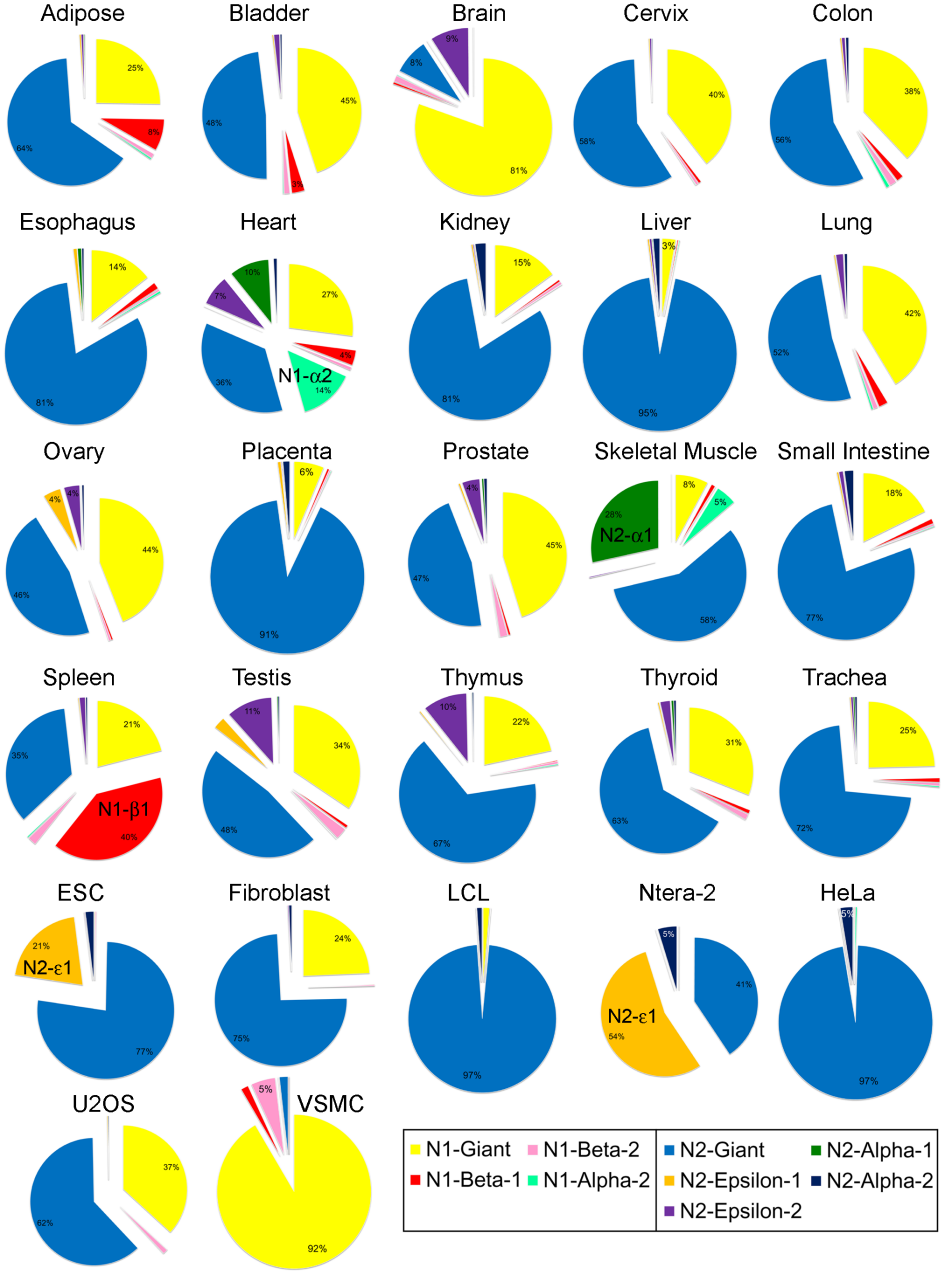
Relative abundance of nesprin isoform mRNAs in human tissues and cell lines. Exploded pie charts show the relative abundance of nesprin isoform mRNA, relative to total nesprin-1 and nesprin-2 mRNA in cDNA preparations from 20 human tissues and 7 human cell lines.

For nesprin-1, we found significant expression, in at least one tissue, of nesprin-1-beta-1 and nesprin-1-alpha-2 (nesprin-1-alpha-1 was not detected and nesprin-1-beta-2 was present at very low levels). For nesprin-2, we consistently found epsilon-1, epsilon-2, alpha-1 and alpha-2 at significant levels (nesprin-2-beta-1 was barely detectable, while nesprin-2-gamma and nesprin-2-beta-2 were present at only low levels). Mean Relative Expression values (±SD) of the nesprin-1 and nesprin-2 isoforms for the 27 cDNA samples are shown in Tables S3 and S4 in File S1. These values are shown as bar charts for selected isoforms (Figs. 2 and 3). The relative abundance of all the different nesprin isoforms are summarised as exploded pie-charts for the 20 human tissues and 7 cell lines (Fig. 4). These results are described in detail under sub-headings below.

### Giant Isoforms, Especially Nesprin-2-giant, are the Dominant Nesprins in most Cells and Tissues

The two giant isoforms accounted for more than 80% of the nesprin mRNA in most of the 20 human tissues, except cardiac muscle (63%), skeletal muscle (66%) and spleen (56%), which were particularly rich in shorter isoforms (Fig. 4). ESC (embryonic stem cells) and Ntera-2 (embryonic teratocarcinoma) cells also contained less than 80% giant isoforms (Fig. 4). There was more nesprin-2 mRNA than nesprin-1 mRNA in most cells and tissues (Fig. 2A), the notable exceptions being brain (81% nesprin-1-giant) and VSMC (vascular smooth muscle cells: 92% nesprin-1-giant). Nesprin-1 mRNA was almost undetectable in ESC, Ntera-2 and HeLa cells (Fig. 4 and Tables S3 and S4 in File S1).

### Nesprin-2-epsilon-1 is Expressed in Early Embryonic Cells, While Nesprin-2-epsilon-2 is Produced in Several Adult Tissues, Including Heart

**Figure 5:**
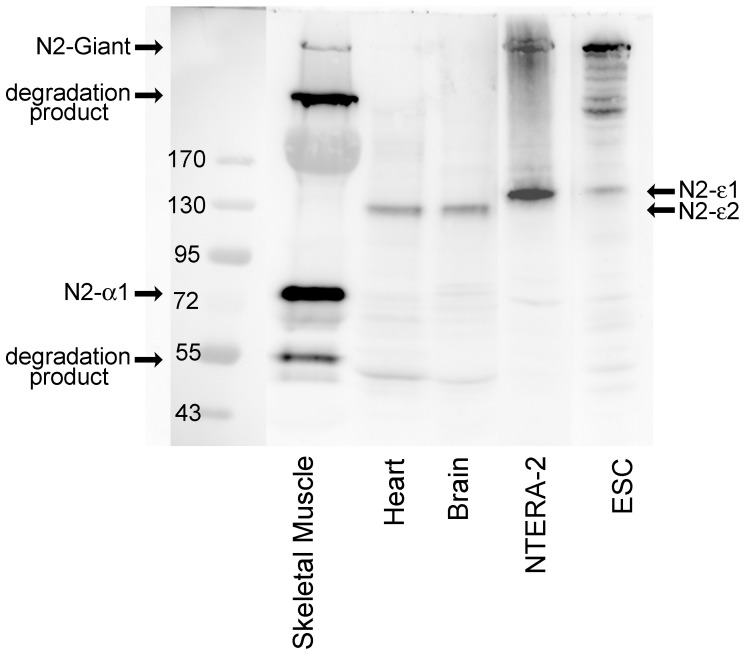
Evidence at the protein level for nesprin-2-epsilon-1 in Ntera-2 and ESC cells and for nesprin-2-epsilon-2 in heart and brain. Western blot for nesprin-2 in human tissues and cell lines using antibody against the C-terminal region of nesprin-2 (MANNES2A 11A3). Bands of approximate size of nesprin-2-epsilon-1 (122 kD) were detected in Ntera-2 and ESC and bands the size of nesprin-2-epsilon-2 (103 kD) were detected in brain and heart. Nesprin-2-alpha-1 was observed in skeletal muscle, but epsilon isoforms were not detected. Absence of mRNA (see Table S4 in File S1), indicates that putative nesprin-2-gamma (377 kD) and nesprin-2-alpha-2 (47 kD) bands on the skeletal muscle western blot, are likely to be degradation products.

We showed in an earlier study [26] that nesprin-2-epsilon-1 mRNA and protein (122 kD) is expressed in Ntera-2 cells and ovary, but not in other tissues, and suggested that it might be an early embryonic isoform. In addition to Ntera-2 and ovary, we now show significant expression of epsilon-1 in ESC which supports the view that it may have a function in early development. Two nesprin-2-epsilon isoforms were predicted by bioinformatics studies [14] and we now show that epsilon-2 mRNA is absent from Ntera-2 and ESC (Figs. 3A and 4), but is expressed at significant levels in heart, brain, thymus, thyroid, prostate, testis and ovary (Figs 3A and 4). The production of epsilon-2 protein (103 kD) by heart and brain is confirmed by western blot, which showed a lower Mr band than the epsilon-1 (122 kD) in ESC and Ntera-2 cells (Fig. 5). Although low levels of nesprin-2-epsilon-2 mRNA were present in skeletal muscle (10% of brain levels), protein was not detected on western blots, possibly because of the sensitivity of the method. Ovarian tissue contains both epsilon-1 and epsilon-2 mRNAs but the total RNA preparation will contain mRNA from a number of “adult” cell types, as well as “embryonic” oocytes. Until specific antibodies to distinguish the two isoforms become available, it is not possible to investigate the exact cellular location of nesprin-2-epsilon-1 by immuno-localisation.

### The Short Alpha Isoforms of both Nesprins are Expressed Mainly in Cardiac and Skeletal Muscle, the two Affected Tissues in Emery-Dreifuss Muscular Dystrophy

Cardiac and skeletal muscles are unusual in expressing, in addition to both giant forms, significant amounts of mRNA for nesprin-1-beta-1, nesprin-1-alpha-2 and nesprin-2-alpha-1 (Fig. 4). Cardiac muscle differs in producing substantial amounts of nesprin-2-epsilon-2, which is virtually absent from skeletal muscle (Figs. 3A and 4). The seven cell lines studied expressed giant forms almost exclusively, except for the large amounts of nesprin-2-epsilon-1 in Ntera-2 and ESC (Figs. 3A and 4) and the presence of nesprin-1-beta-1/beta-2 in VSMC (Fig. 4 and Table S3 in File S1). Relative expression of the two nesprin genes varied greatly between cell lines, with HeLa expressing nesprin-2 and VSMC expressing nesprin-1 almost exclusively (Fig. 4). This suggests that any non-specialised functions can be performed by either of the two nesprins.

### Use of mRNA Studies to Distinguish True Isoforms from Degradation Products

In an earlier study [27], we suggested that skeletal muscle contains nesprin-2-gamma (377 kD) and nesprin-2-alpha-2 (47 kD), in addition to giant and nesprin-2-alpha-1 isoforms, and all four bands (plus a “ghost” band of myosin at 200 kD) can be seen clearly in Fig. 5. However, mRNAs for gamma and nesprin-2-alpha-2 are barely detectable in skeletal muscle (Fig. 3B and Table S4 in File S1), suggesting that giant (792 kD) and alpha-1 (60 kD) are the only significant isoforms of nesprin-2 produced by skeletal muscle and the two bands at ca. 400 kD and ca. 50 kD are likely degradation products of higher Mr isoforms. The alternative possibility that these lower Mr bands are due to cross-reactions of the antibody with non-nesprin proteins has been eliminated by the use of monoclonal antibodies (mAbs) against different epitopes in the C-terminal region [27]. Although nesprin-2-alpha-2 mRNA was absent from skeletal muscle, we did detect significant amounts in some cell lines and tissues, notably kidney (Fig. 3B).

### Nesprin-1-beta-1 Protein is Produced in Spleen

**Figure 6:**
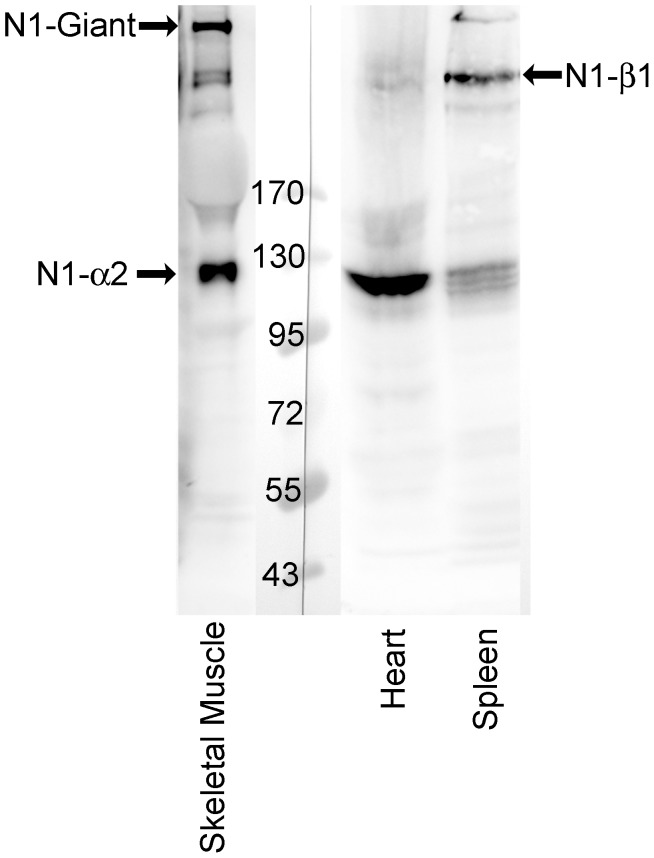
Evidence at the protein level for nesprin-1-beta-1 in spleen and for nesprin-1-alpha-2 in cardiac and skeletal muscle. Western blot for nesprin-1 in tissues using antibody against the C-terminal region of nesprin-1 (MANNES1E 8C3). A band the size of nesprin-1-beta-1 (383 kD) was detected in spleen and bands of nesprin-1-alpha-2 (111 kD) were detected in skeletal muscle and heart.

Since the qPCR data showed unusually high levels of nesprin-1-beta-1 in spleen (Figs. 3B and 4), we performed a western blot on an extract of pig spleen (Fig. 6). Consistent with qPCR, a band of the expected molecular weight for the nesprin-1-beta-1 isoform was found in spleen. In heart, which has 10-fold lower levels of nesprin-1-beta-1 mRNA, this band was barely detectable. Heart does have a prominent band of nesprin-1-alpha-2 protein (Fig. 6), as predicted from qPCR, but the triplet protein band in this region of the spleen blot is unlikely to be this isoform, since the mRNA for nesprin-1-alpha-2 is not expressed in spleen. This again illustrates the value of running qPCR alongside western blots to identify true nesprin isoforms.

### Alternative Splicing of the Highly-conserved DV23 Exon of Nesprins is Highly Variable between Tissues

**Figure 7:**
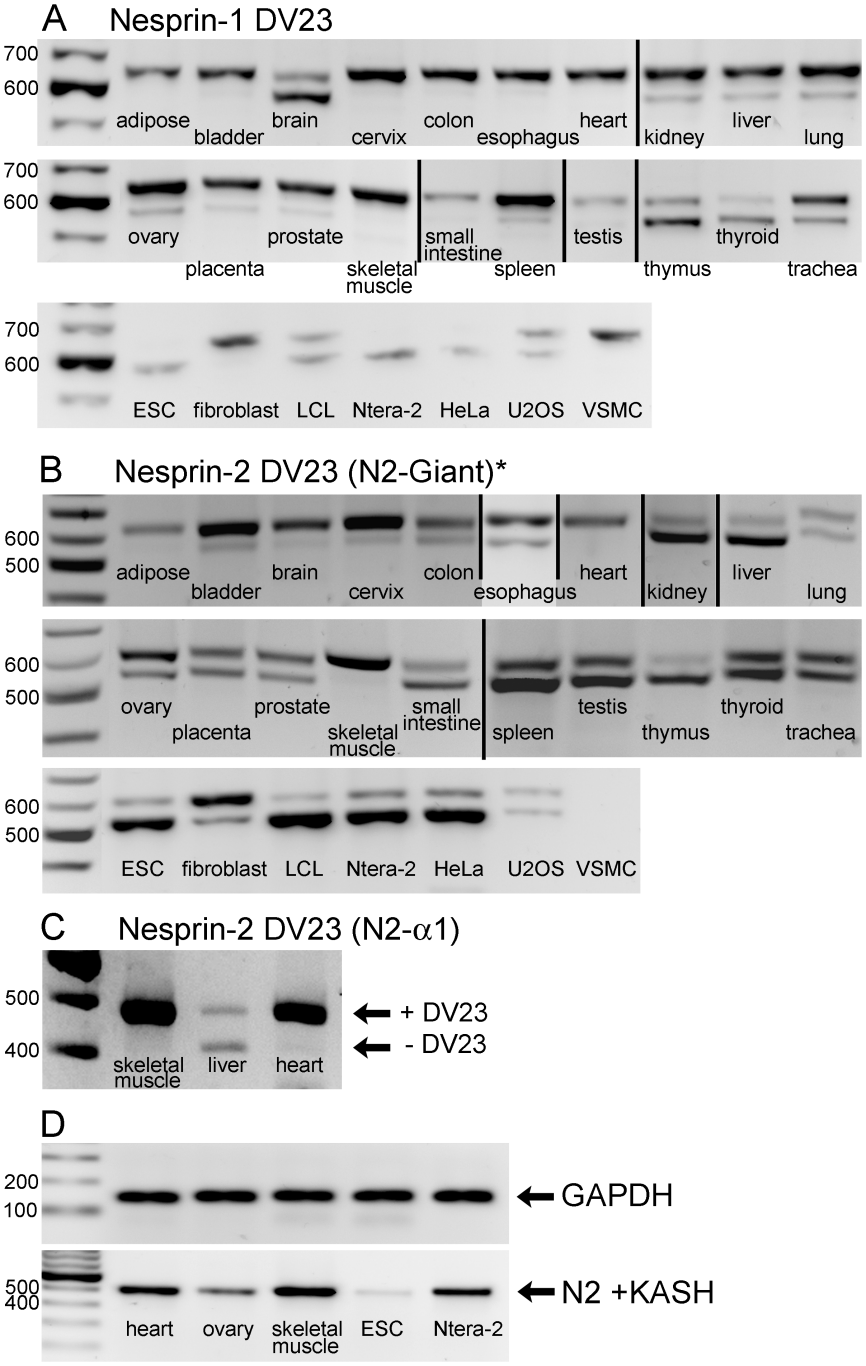
Tissue-dependent alternative splicing of the highly conserved DV23 exon of nesprin-1 and nesprin-2 and the KASH domain of nesprin-2. Products of conventional PCR were separated on agarose gels. The 27 human cDNA samples, as shown in Table 1, were used in A and B. For DV23, the upper band contained the DV23 region and the lower band lacked the DV23 region. Specific primer pairs spanned: (A) The nesprin-1 DV23 region of nesprin-1-giant and other nesprin-1 isoforms, (B) the nesprin-2 DV23 region of nesprin-2-giant (which included other nesprin-2 isoforms except nesprin-2-alpha-1) and (C) the nesprin-2 DV23 region of only the nesprin-2-alpha-1 isoform. Vertical black lines indicate images from different gels that have been compiled. (D) PCR was used to detect the presence of nesprin-2 KASH, along with GAPDH control in 5 cDNA samples.

To avoid the implication that the sequence is absent or deleted, we have used the term “DV23” to describe the 69 bp exon sequence, described by Simpson and Roberts [14] as “ΔSR”. The product of DV23 contains 23 amino acids, beginning with Aspartic Acid (D) and Valine (V). Bioinformatics showed that this exon is highly conserved across species and is present in both nesprin-1 and nesprin-2, suggesting that this sequence has an important function [14]. Fig. 7 shows PCR across the DV23 exon of nesprin-1 and nesprin-2, to determine the relative amounts of its inclusion by alternative splicing in different tissues. The nesprin-1 PCR primers detected DV23 in all nesprin-1 isoform mRNAs (Fig. 7A). The nesprin-2 primers (Fig. 7B) did not amplify DV23 contained in the nesprin-2-alpha-1 isoform, so the results refer mainly to nesprin-2-giant, unless there was significant expression of other isoforms, such as epsilon. There were high levels of inclusion of DV23 for both nesprins in cardiac and skeletal muscle. Using a primer pair specific for nesprin-2-alpha-1, inclusion of DV23 in nesprin-2-alpha-1 was also >95% in cardiac and skeletal muscle (Fig. 7C).

Inclusion of DV23 varied from 100% in some tissues (including cardiac muscle) to <25% in others, and even lower in some transformed cell lines (Fig. 7 and Table 1). Generally, inclusion of DV23 in nesprin-2 was similar to, or higher than, in nesprin-1 for most tissues, but brain was a notable exception with 89% inclusion in nesprin-2 but only 31% in nesprin-1 (Table 1).

**Table 1:** Nesprin DV23 inclusion in cDNA from tissues and cells.

Sample Number	cDNA	Nesprin-1 DV23 inclusion (%)	Nesprin-2 DV23 inclusion (%)	
			Except nesprin-2-alpha-1	Only nesprin-2-alpha-1
1	Adipose	100	100	
2	Bladder	97	84	
3	Brain	31	89	
4	Cervix	100	87	
5	Colon	100	71	
6	Esophagus	99	73	
7	Heart	100	100	100
8	Kidney	81	27	
9	Liver	80	22	53*
10	Lung	83	61	
11	Ovary	82	71	
12	Placenta	86	56	
13	Prostate	88	63	
14	Skeletal Muscle	94	94	100
15	Small Intestine	83	38	
16	Spleen	87	39	
17	Testis	76	43	
18	Thymus	30	24	
19	Thyroid	24	49	
20	Trachea	68	52	
21	ESC	24	17	
22	Fibroblast	92	75	
23	LCL	47	9	
24	Ntera-2	10	19	
25	HeLa	23	19	
26	U2OS	65	53	
27	VSMC	95	Not detected	

*It was necessary to perform PCR on the PCR product to obtain the bands with liver cDNA using nesprin-2-alpha-1 primers.

**Figure 8:**
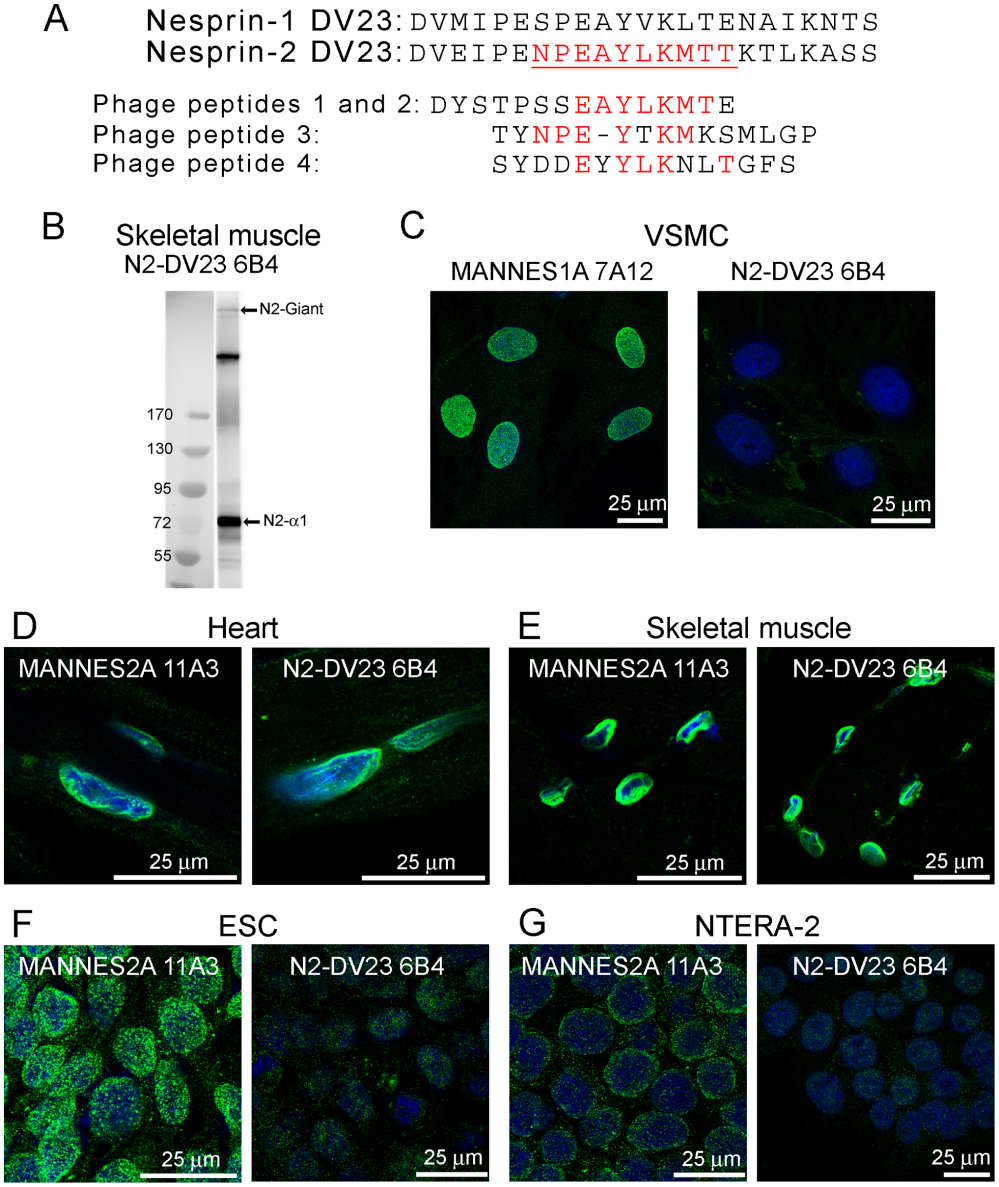
Immunolocalisation of isoforms lacking the DV23 region and/or the KASH domain. (A) Alignment of the 23 amino acid sequences of the DV23 exon of nesprin-1 and nesprin-2. The nesprin-2 DV23 peptide was the immunogen for mAb production. The epitope of the mAbs was mapped to the sequence shown in red and underlined. Sequences of the four 15-mer peptides pulled out of the phage-display peptide library are shown aligned below the nesprin-2 DV23 sequence, with matching amino acids shown in red. (B) The mAb N2-DV23 6B4 recognised bands of nesprin-2-giant and nesprin-2-alpha-1 in western blot of skeletal muscle (the intermediate band is a likely degradation product of nesprin-2-giant). (C) The 6B4 mAb against nesprin-2 DV23 does not cross-react with nesprin-1 DV23. VSMC (which contain nesprin-1 with DV23 but no nesprin-2) showed nuclear staining for nesprin-1 with MANNES1A but not with 6B4. Immunofluorescent staining with mAbs MANNES2A and N2-DV23 6B4 was equally strong on both cardiac muscle nuclear rim (D) and skeletal muscle nuclear rim (E). However, MANNES2A was much stronger than N2-DV23 6B4 on both ESC (F) and Ntera-2 cells (G), since the DV23 exon is mainly excluded in these cells (see Fig. 7B). Furthermore, the MANNES1A mAb is largely nucleoplasmic in ESC, which may be because the nesprin-2 KASH domain is also largely excluded in this cell type (see Fig. 7D).

Fig. 8A shows the 23-amino-acid sequence of DV23 in both nesprins. It is quite highly-conserved, even between the two human nesprin genes (57% identity). We produced a panel of 8 mAbs against the DV23 sequence of nesprin-2 using a peptide-conjugate as immunogen and mapped their epitopes using a phage-displayed random peptide library. All 8 mAbs recognised four peptides, which are shown aligned with each other and with the nesprin-2 DV23 sequence in figure 8A. Common amino acids are shown in red and the mapped epitope is underlined in Fig. 8A. PCR showed strong inclusion of the nesprin-2 DV23 exon in nesprin-2-giant and nesprin-2-alpha-1 in skeletal muscle, which was confirmed by western blot with one of these mAbs, N2-DV23 6B4 (Fig. 8B). Six of the 10 amino-acids in the epitope of mAb N2-DV23 6B4 are identical in nesprins -1 and -2, so it was necessary to demonstrate specificity of the mAb for nesprin-2 experimentally. Fig. 8C shows that mAb N2-DV23 6B4 does not stain the nuclear membrane in vascular smooth muscle cells; VSMC do not have nesprin-2 although they do express high levels of nesprin-1 containing DV23, so confirming the specificity of this antibody for nesprin-2. Fig. 8D-G shows that the same mAb (N2-DV23 6B4) stained the nuclear rim as strongly as mAbs against total nesprin-2 in tissues where DV23 is mainly included (heart and skeletal muscle), but shows much weaker nuclear staining in cells where nesprin-2 DV23 is largely excluded (ESC and Ntera-2). This confirms the mRNA results for DV23 at the protein level.

### Nesprin-2 Lacking the KASH Domain Locates in the Nucleoplasm of Embryonic Stem Cells

The nesprin-2 mAb (MANNES2A), showed only nuclear rim staining in heart and skeletal muscle sections (Fig. 8D, E). This mAb in ESC showed an intense nucleoplasmic speckle-like distribution (Fig. 8F) and in Ntera-2 there was some cytoplasmic stain (Fig. 8G). PCR for nesprin-2 KASH indicated that most of the nesprin-2 mRNA in ESC, lacked the KASH domain (Fig. 7D). Heart and skeletal muscle appeared to contain the highest levels of nesprin-2 KASH, with ovary and Ntera-2 also positive for KASH (Fig. 7D). These results in combination indicate that the nucleoplasmic, speckle-like distribution of nesprin-2 in ESC is attributable to absence of the KASH domain.

## Discussion

### Support for the different Isoforms of Nesprin-1 and Nesprin-2

This study differs from earlier studies, first of all in using qPCR with internal standards to determine the relative levels of all known KASH domain isoforms (and their KASH-less splice variants), of both nesprins-1 and -2, and secondly, in combining mRNA (qPCR) and protein (western blot) data to distinguish true isoforms from degradation products at the protein level. We have concluded that the main isoforms expressed from *SYNE1* are nesprin-1-giant (1008 kD), nesprin-1-alpha-2 (111 kD) and, at lower levels, two forms of nesprin-1-beta (383 kD and 370 kD), but nesprin-1-alpha-1 mRNA was barely detectable. The main isoforms from *SYNE2* were nesprin-2-giant (792 kD), two forms of nesprin-2-epsilon (103 kD and 122 kD) and two forms of nesprin-2-alpha (60 kD and 47 kD). Nesprin-2-gamma and nesprin-2-beta-2 were present at only very low levels, while nesprin-2-beta-1 was barely detectable. It is perhaps surprising that we were able to amplify nesprin-1-alpha-1 and nesprin-2-beta-1 by conventional PCR but were unable to determine efficiency of qPCR, using the same primer pairs. In this respect, it has been reported that conventional PCR and qPCR may perform differently and that conventional PCR may have greater analytical sensitivity compared with qPCR [28]. The conclusions are in excellent agreement with the bioinformatics studies of Simpson and Roberts [14], which found “evidence against a biological role” for both nesprin-1-alpha-1 and nesprin-2-beta-1 and “strong support for the biological relevance” of nesprin-1-alpha-2, both nesprin-2-epsilons and both nesprin-2-alphas. They had insufficient sequence data to make reliable predictions about other beta and gamma isoforms [14]. It seems as though only the giant isoforms of nesprins are essential for basic cell functions, since some of the cell lines lack shorter isoforms. Similarly, some cell lines showed almost exclusive expression from only one of the two nesprin genes.

The tissue distribution data on nesprin-2-giant mRNA agree broadly with the hybridization array data of Zhen et al. [3] with highest expression in kidney, liver and thyroid and low levels in brain, except that we found significant amounts in HeLa and other cell lines and only average levels in spleen. The unusually high levels of nesprin-1-beta-1 mRNA and protein in spleen are consistent with previous northern blot analysis [2] and show the importance of avoiding generalisations about nesprins based on a limited range of cells or tissues. Relative to GAPDH and beta-actin, overall nesprin mRNA levels were high in kidney and thyroid, but low in brain, bladder, thymus and all seven cell lines. Tissues are not homogeneous and contain a variety of cell types, including vascular cells and blood/lymph cells. It is possible, therefore, that the high levels of nesprin-1-beta-1 mRNA and protein in spleen can be traceable to one particular cell type. Similarly, the expression of both nesprin-2-epsilon-1 and epsilon-2 in ovary may occur in different cell types in that tissue. Future availability of isoform-specific mAbs should enable us to answer this question in the long-term using immunolocalisation microscopy. The detection of nesprin-2-epsilon-1 only in cells of embryonic origin suggests that it may occur in germ cells of ovary, but this has not yet been confirmed. The question of whether cardiac nesprin-2-epsilon-2 expression is attributable to any particular cell type in the heart could also be answered with epsilon-2-specific mAbs.

### Validation of Western Blots by qPCR

We have also been able, where possible, to reconcile the mRNA data with protein data from western blots, although available antibodies are only able to detect isoforms produced at reasonably high levels. The main problem in identifying bands on western blots as authentic short forms of nesprin is the possibility that lower Mr bands might be due to proteolysis of a larger isoform, such as the “giant” form. This is especially problematical when a larger isoform is present in great abundance. Large cytoskeletal proteins, like dystrophin and nesprins, are especially sensitive to proteolysis and even when precautionary extraction methods are used (see Methods), human tissues with no degradation are difficult to obtain. Although human extracts and ready-made blots covering a range of tissues are available commercially, we have found them too dilute for detection of nesprins with our mAbs, although they do work with antibodies against more abundant proteins, such as GAPDH (unpublished observations). True isoforms can in theory be identified using antibodies against isoform-specific amino-acid sequences (usually at the amino-terminus) but such antibodies are not yet available. The availability of qPCR data does throw some light on this problem. Protein levels normally reflect levels of the corresponding mRNA, though differences in protein stability or turnover may complicate the relationship. These considerations suggest that, in skeletal muscle, the only KASH domain proteins produced in significant amounts are the two giant forms plus nesprin-2-alpha-1 (60 kD) and nesprin-1-alpha-2 (111 kD). Very small amounts of nesprin-1-beta-1 and nesprin-2-epsilon-2 mRNA were also detected in skeletal muscle, but the putative nesprin-2-gamma (377 kD) and nesprin-2-alpha-2 (47 kD) proteins identified on western blots [27] are likely degradation products (see also Fig. 5).

### “DV23” Splice Variants

Although this study has clarified the major KASH-containing nesprin isoforms that are expressed, other studies have shown that alternative splicing can produce additional variants within this basic framework. We therefore studied splicing of the “DV23” exon, which has predicted functional importance [14] and is located near the N-terminal of nesprin-2-alpha-1. DV23 was largely included in nesprin-2-giant mRNA from skeletal muscle, heart, brain and several other tissues, but largely excluded in liver, kidney and thymus. The DV23 exon was also included in nesprin-2-alpha-1 mRNA in cardiac and skeletal muscle, which was also seen in an earlier report of total nesprin-2 [1]. Recently, many alternative start and termination sites throughout the nesprin-1 and nesprin-2 genes have been identified, giving the possibility of multiple short isoform variants [29].

### Is Emery-Dreifuss Muscular Dystrophy caused by Defects in a Specific Function of the Short Nesprin Isoforms in Cardiac and Skeletal Muscle?

The observation, from qPCR and western blot, that the short alpha isoforms, nesprin-1-alpha-2 and nesprin-2-alpha-1, are expressed almost exclusively in cardiac and skeletal muscle is consistent with previous northern analysis [1], [2] and RT-PCR [4] and extends these earlier results to a wider range of tissues. Cardiac and skeletal muscles are also the only two tissues affected in EDMD. All the known mutations in nesprins that are associated with EDMD or dilated cardiomyopathy, lie within the alpha isoform sequences [17]–[19]. The tissue-specific distribution of the alpha isoforms, and the location of pathogenic nesprin mutations within them, raises the possibility that alpha isoforms have some specific function that is not shared by the full-length “giant” forms. Loss of this function may then be responsible for the pathogenesis of EDMD and dilated cardiomyopathy.

One current hypothesis is that short isoforms of nesprin locate to the inner nuclear membrane, where they can interact directly with emerin and lamin A/C, mutations in which also cause EDMD. The idea of loss of function of a complex of emerin, lamin A/C and nesprins as a unifying cause of EDMD is an attractive one, but no function requiring all three proteins has yet been identified. When they are localised at the outer nuclear membrane, giant nesprins can interact only indirectly with lamin A/C, via SUN proteins at the inner nuclear membrane. Although giant isoforms may locate to the inner membrane during assembly of the nuclear envelope after mitosis, such large proteins may be unable to cross the assembled nuclear envelope while still membrane-associated [30]. However, nesprin isoforms without KASH and transmembrane domains have been described and these “non-membrane” proteins can enter the nucleus through the nuclear pores. When transfected, isoforms of nesprin-2 that lack a KASH domain have been shown to form nuclear complexes and colocalise with promyelocytic leukemia protein (PML) bodies [31]. In this respect, we have shown that most of the nesprin-2 in ESC lacks the KASH domain and is largely located in the nucleoplasm with a speckle-like appearance. Nesprin-2-giant has also been found with centromeric and heterochromatic sequences inside the nucleus [32]. Immuno-gold EM studies using a keratinocyte cell line, showed nesprin-2-giant at both the outer and inner nuclear membranes [33]. One consequence of the presence of giant isoforms, with or without KASH domains, inside the nucleus is that formation of complexes with emerin/lamin A/C is not an exclusive function of short isoforms.

Nearly all the pathogenic mutations in human nesprins known so far are autosomal dominant mutations in nesprin-1. Nesprin-1-giant does appear to have an essential function in myonuclear positioning. A mouse knockout of the nesprin-1 KASH domain had myonuclear localisation defects that were not shared by the corresponding nesprin-2 KASH knockout mouse and disruption of nesprin-1 KASH or nesprin-2 KASH did not affect viability or fertility [34]. Knockout of the actin-binding region of nesprin-2 generated a mouse model with complete loss of nesprin-2-giant [35]. These mice had an increased thickness of epidermis and showed defective wound healing, but were otherwise very similar to wild type mice and did not have an EDMD phenotype [32], [35]. Similarly, mice with a knockout of the C-terminal spectrin repeat region of nesprin-1 which resulted in ablation of most isoforms of nesprin-1 showed defects in positioning and anchorage of nuclei in skeletal muscle, but had normal heart function and did not display a phenotype similar to EDMD [36]. The bioinformatic study of Simpson and Roberts [14] found nesprin-2-alpha-1 only in human, but not in mouse, whereas nesprin-1-alpha-2 and both epsilon isoforms of nesprin-2 were present in human and mouse. Knockout of either *Sun1* alone or double-knockout of both *Sun1* and *Sun2* also caused defects in myonuclear positioning, rather than an EDMD-like phenotype [37]. A mouse model in which the KASH domain of nesprin-1 was replaced with an unrelated sequence did have an EDMD-like phenotype [38], consistent with a dominant-negative effect of expression of a mutant protein. It would seem that some, but not all, functions of nesprins can be performed by either nesprin-1 or nesprin-2, since mice with a double-knockout of both nesprins have respiratory failure and die shortly after birth [34]. The two giant nesprins do not form obligatory complexes together, since nesprin-1-giant and nesprin-2-giant do not co-localise in EDMD skin fibroblasts without emerin [27]. If giant isoforms of the two nesprins do not interact with each other, short isoforms are unlikely to form heterodimers either, although nesprin-1-alpha does form homodimers, which bind directly to emerin and lamin A [12].

The presence of nesprin mutations that are likely to be pathogenic and the muscle-specific distribution of the alpha isoforms, raises the possibility that alpha isoforms have some specific function that is not shared by the full-length “giant” forms. Loss of this function may then be responsible for the pathogenesis of EDMD and dilated cardiomyopathy.

## Materials and Methods

### Ethics Statement

This study has been approved by the Robert Jones and Agnes Hunt Orthopaedic Hospital Research Committee. Human biopsies were obtained following written informed consent using protocols approved by Hammersmith Hospital and the University of Cambridge. The monoclonal antibody production protocol was performed with approval of the Keele University Animal Welfare and Ethical Review Body.

### Cell Culture

Ntera-2 (pluripotent neuroectodermal human testicular embryonic teratocarcinoma cell line, gift from Peter Andrews, Sheffield University [39]), LCL (lymphoblastoid cell line [40]), HeLa (human epithelial carcinoma cell line [41]), fibroblasts (human fibroblasts established in culture from skin biopsy [42]), and U2OS (human osteosarcoma epithelial cell line, obtained from American Tissue Culture Collection (ATCC)), were grown in DMEM with 10% fetal bovine serum and antibiotics and VSMC (vascular smooth muscle cells [43]) were grown in Medium 199 with 20% fetal bovine serum and antibiotics. The H9 Embryonic Stem Cell (ESC) line (gift from Rachel Oldershaw, Newcastle University [44]) was grown in StemPro hESC Serum-Free Medium (Gibco) on fibronectin-coated tissue culture wells. Clumps of cells were dissociated with TrypLE Express (Gibco).

### RT-PCR and qPCR

The First Choice Human Total RNA Survey Panel (ABI Ambion, Austin, Texas) was used as a source of RNA from 20 adult human tissues. Total RNA was prepared from cultured cells using RNeasy Plus Mini Kit (Qiagen) and quantified using a NanoDrop ND-1000 spectrophotometer. Total RNA (2 μg in a 20 μL reaction) was reverse transcribed using SuperScript VILO cDNA Synthesis Kit (Applied Biosystems) and then diluted 1∶12 with sterile water.

Forward primers for specific short isoforms of nesprin-1 and nesprin-2 were each designed to recognise a unique sequence in the 5′ UTR of the isoform. Primer pairs for the giant isoforms were designed, to minimise amplification of shorter isoforms. Short isoforms containing the N-terminal CH domains have recently been described [29] and we designed primers to avoid these. Primers for nesprin-1-giant span the N-terminal site of nesprin-1-beta-1 and primers for nesprin-2-giant span the N-terminal site of nesprin-2-epsilon-1. Primer sequences are shown in Table S1 in File S1. Primer pairs were tested by conventional PCR (PCR Core Kit, Qiagen), and products confirmed by DNA sequencing (DNA Sequencing and Services, University of Dundee).

Relative quantitative PCR was performed using SYBR green detection in an ABI 7500 Real Time PCR system (Applied Biosystems). Reaction wells contained 10 μL SYBR Select Master Mix (Applied Biosystems), 1.5 μL cDNA, 300 nM Forward and 300 nM Reverse primers in a final volume of 20 μL. For each preparation of cDNA, each target sequence was amplified along with two endogenous controls (Beta-actin and GAPDH). Quantitation of target transcripts relative to the two endogenous reference transcripts were calculated by the 2^-ΔCT^ method [45], . The efficiency of primer pairs for quantitative PCR was determined by making serial dilutions of the cDNA, performing absolute quantitation, plotting C_T_ versus log cDNA dilution, with the slope of the line being used to calculate efficiency [46]. Dissociation curves were obtained to ensure that each primer pair gave a single peak. When an isoform was detected, qPCR was performed at least three times.

Specific primer pairs to span the DV23 regions of nesprin-1, nesprin-2 (all isoforms except nesprin-2-alpha-1) and nesprin-2-alpha-1 are shown in Table S2 in File S1. Specific products were confirmed by sequencing. Where necessary, products were cut from agarose gels and purified (Qiaex II gel extraction kit, Qiagen) prior to sequencing. Upper bands contained DV23 and lower bands lacked DV23. Values for percent inclusion of DV23 were calculated following image analysis of the agarose gels. A forward primer within nesprin-2 KASH and a reverse primer downstream of this were used to detect the presence of the nesprin-2 KASH domain, which was also confirmed by sequencing.

### Hybridoma Production

Peptide: DVEIPENPEAYLKMTTKTLKASSC with Keyhole Limpet Hemocyanin conjugated to the C-terminal (AltaBioscience, Birmingham UK), was used as immunogen for production of monoclonal antibodies against DV23 region of nesprin-2, using the hybridoma method [47]. Epitope mapping of monoclonal antibodies was performed using phage-displayed random peptide libraries in filamentous phage as previously described [48], Briefly, monoclonal antibody mixtures were diluted 1∶50 with Tris-buffered saline (TBS) and immobilised onto sterile 35 mm Petri dishes coated directly with 1 ml of 1∶200 dilution of rabbit-anti-[mouse Ig] in TBS (DAKOs, Denmark). Biopanning was performed using a 15-mer peptide library in phage f88-4, maintained in the K91Kan strain of *E. coli* (G.P. Smith, University of Missouri). Any remaining binding sites on the dishes were blocked using 4% BSA in sterile TBS. A sample of the phage library (10^13^ virions) was pre-incubated in dishes coated with the rabbit anti-mouse antibodies alone to ensure any binding was specific for the target mAbs. Following the first round of biopanning, the bound phage were eluted and amplified by infection of K91Kan *E. coli* cells. Two rounds of biopanning were performed. Individual colonies of the phage-infected cells after the second round were grown on nitrocellulose membrane (BA85) and screened by western blotting to reveal positive clones. Positive clones were subjected to western blotting with individual mAbs from the mixture used for biopanning. After blocking non-specific sites with 5% skimmed milk protein in TBS, membranes were incubated with mAb supernatant (1/100 dilution in TBS). Antibody-reacting clones were visualized following development with biotinylated horse anti-mouse Ig in a Vectastain ABC kit (Vector Labs, Burlingame, CA) and diaminobenzidine substrate (Sigma; 0.4 mg/ml). Phage DNA was purified from positive clones by the phenol/chloroform method and sequenced using primer: 5′-AGTAGCAGAAGCCTGAAGA-3′.

Other monoclonal antibodies used in this study were: MANNES1A 7A12 (for immunofluorescence) and MANNES1E 8C3 (for western blot), which both recognise the C-terminal of nesprin-1; and MANNES2A 11A3 which has an epitope in exon 112 of the C-terminal of nesprin-2 [27].

### SDS-polyacrylamide Gel Electrophoresis and Western Blotting

Cultured cells were extracted in 125 mM Tris pH 6.8, 2% SDS, 5% 2-beta mercaptoethanol, 5% glycerol with protease inhibitors (Sigma P8340 plus 1 mM PMSF). Tissue samples (250 mg/ml) were extracted in: 50 mM Tris pH 6.8, 1% EDTA, 10% SDS, 5% beta mercaptoethanol, 10% glycerol with protease inhibitors. After the addition of bromophenol blue and after boiling, samples were subjected to SDS-PAGE using 4 to 12% polyacrylamide gels and transferred to nitrocellulose membranes (Protan BA85, Whatman). After blocking non-specific sites with 5% skimmed milk protein, membranes were incubated with monoclonal antibody (1/50), followed by washing and incubation with peroxidase-labelled rabbit anti-mouse immunoglobulins (1/1000, Dako, Denmark). Antibody reacting bands were visualized with West Femto chemiluminescent detection system (Pierce, Thermo Scientific).

### Immunofluorescence Microscopy

Immunohistochemistry was performed on unfixed cryostat sections and also on cells that had been cultured on coverslips, fixed with 50∶50 acetone-methanol and washed with PBS. Monoclonal antibodies in culture supernatants were diluted 1∶3 in PBS and incubated on the specimen for 1 hour. Following washing, incubation was continued with 5 μg/ml goat anti-mouse ALEXA 488 (Molecular Probes, Eugene, Oregon, USA) secondary antibody diluted in PBS containing 1% horse serum, 1% fetal bovine serum and 0.1% BSA, for 1 hour. DAPI (diamidino phenylindole at 200 ng/ml) was added for the final 10 minutes of incubation to counterstain nuclei before mounting in Hydromount (Merck). Sequential confocal scans were performed with a Leica TCS SP5 spectral confocal microscope (Leica Microsystems, Milton Keynes, UK).

## Supporting Information

File S1 Contains Table S1, Quantitative PCR primer pairs. Table S2, PCR primer pairs used to span the 69 bp DV23 regions of nesprin-1 and nesprin-2 and to amplify from within the KASH region of nesprin-2. Table S3, Relative expression (RE) of nesprin-1 (N1) isoforms in human tissues and cultured cells. Table S4, Relative expression (RE) of nesprin-2 (N2) isoforms in human tissues and cultured cells.
(PDF)

## References

[1] Apel ED, Lewis RM, Grady RM, Sanes JR (2000) Syne-1, a dystrophin- and klarsicht-related protein associated with synaptic nuclei at the neuromuscular junction. J Biol Chem 275: 31986–31995. 10878022 10.1074/jbc.M004775200

[2] Zhang Q, Skepper JN, Yang F, Davies JD, Hegyi L, et al (2001) Nesprins: a novel family of spectrin-repeat-containing proteins that localize to the nuclear membrane in multiple tissues. J Cell Sci 114: 4485–98. 11792814 10.1242/jcs.114.24.4485

[3] Zhen YY, Libotte T, Munck M, Noegel AA, Korenbaum E (2002) NUANCE, a giant protein connecting the nucleus and actin cytoskeleton. J Cell Sci 115: 3207–3222. 12118075 10.1242/jcs.115.15.3207

[4] Zhang Q, Ragnauth CD, Skepper JN, Worth NF, Warren DT, et al (2005) Nesprin-2 is a multi-isomeric protein that binds lamin and emerin at the nuclear envelope and forms a subcellular network in skeletal muscle. J Cell Sci 118: 673–687. 15671068 10.1242/jcs.01642

[5] Mislow JM, Kim MS, Davis DB, McNally EM (2002) Myne-1, a spectrin repeat transmembrane protein of the myocyte inner nuclear membrane, interacts with lamin A/C. J Cell Sci 115: 61–70. 11801724 10.1242/jcs.115.1.61

[6] Padmakumar VC, Abraham S, Braune S, Noegel AA, Tunggal B, et al (2004) Enaptin, a giant actin-binding protein, is an element of the nuclear membrane and the actin cytoskeleton. Exp Cell Res 295: 330–339. 15093733 10.1016/j.yexcr.2004.01.014

[7] Sosa BA, Rothballer A, Kutay U, Schwartz TU (2012) LINC complexes form by binding of three KASH peptides to domain interfaces of trimeric SUN proteins. Cell 149: 1035–1047. 22632968 10.1016/j.cell.2012.03.046 PMC3383001

[8] Zhou Z, Du X, Cai Z, Song X, Zhang H, et al (2012) Structure of Sad1-UNC84 homology (SUN) domain defines features of molecular bridge in nuclear envelope. J Biol Chem 287: 5317–5326. 22170055 10.1074/jbc.M111.304543 PMC3285312

[9] Lombardi ML, Lammerding J (2011) Keeping the LINC: the importance of nucleocytoskeletal coupling in intracellular force transmission and cellular function. Biochem Soc Trans 39: 1729–1734. 22103516 10.1042/BST20110686 PMC4589539

[10] Rajgor D, Shanahan CM (2013) Nesprins: from the nuclear envelope and beyond. Expert Rev Mol Med 15: e5. 23830188 10.1017/erm.2013.6 PMC3733404

[11] Haque F, Mazzeo D, Patel JT, Smallwood DT, Ellis JA, et al (2010) Mammalian SUN protein interaction networks at the inner nuclear membrane and their role in laminopathy disease processes. J Biol Chem 285: 3487–3498. 19933576 10.1074/jbc.M109.071910 PMC2823409

[12] Mislow JM, Holaska JM, Kim MS, Lee KK, Segura-Totten M, et al (2002) Nesprin-1alpha self-associates and binds directly to emerin and lamin A in vitro. FEBS Lett 525: 135–140. 12163176 10.1016/s0014-5793(02)03105-8

[13] Pare GC, Easlick JL, Mislow JM, McNally EM, Kapiloff MS (2005) Nesprin-1alpha contributes to the targeting of mAKAP to the cardiac myocyte nuclear envelope. Exp Cell Res 303: 388–399. 15652351 10.1016/j.yexcr.2004.10.009

[14] Simpson JG, Roberts RG (2008) Patterns of evolutionary conservation in the nesprin genes highlight probable functionally important protein domains and isoforms. Biochem Soc Trans 36: 1359–1367. 19021556 10.1042/BST0361359

[15] Wilhelmsen K, Litjens SH, Kuikman I, Tshimbalanga N, Janssen H, et al (2005) Nesprin-3, a novel outer nuclear membrane protein, associates with the cytoskeletal linker protein plectin. J Cell Biol 171: 799–810. 16330710 10.1083/jcb.200506083 PMC2171291

[16] Roux KJ, Crisp ML, Liu Q, Kim D, Kozlov S, et al (2009) Nesprin 4 is an outer nuclear membrane protein that can induce kinesin-mediated cell polarization. Proc Natl Acad Sci USA 106: 2194–2199. 19164528 10.1073/pnas.0808602106 PMC2650131

[17] Zhang Q, Bethmann C, Worth NF, Davies JD, Wasner C, et al (2007) Nesprin-1 and -2 are involved in the pathogenesis of Emery Dreifuss muscular dystrophy and are critical for nuclear envelope integrity. Hum Mol Genet 16(23): 2816–2833. 17761684 10.1093/hmg/ddm238

[18] Puckelwartz MJ, Kessler EJ, Kim G, Dewitt MM, Zhang Y, et al (2010) Nesprin-1 mutations in human and murine cardiomyopathy. J Mol Cell Cardiol 48: 600–608. 19944109 10.1016/j.yjmcc.2009.11.006 PMC2837775

[19] Li C, Zhou B, Rao L, Shannahan CM, Zhang Q (2011) Novel nesprin-1 mutations in human dilated cardiomyopathy. Nuclear envelope disease and chromatin organization meeting, Cambridge. Abstract P028.

[20] Bione S, Maestrini E, Rivella S, Mancini M, Regis S, et al (1994) Identification of a novel X-linked gene responsible for Emery-Dreifuss muscular dystrophy. Nat Genet 8: 323–327. 7894480 10.1038/ng1294-323

[21] Bonne G, Di Barletta MR, Varnous S, Bécane HM, Hammouda EH, et al (1999) Mutations in the gene encoding lamin A/C cause autosomal dominant Emery-Dreifuss muscular dystrophy. Nat Genet 21: 285–288. 10080180 10.1038/6799

[22] Meinke P, Nguyen TD, Wehnert MS (2011) The LINC complex and human disease. Biochem Soc Trans 39: 1693–1697. 22103509 10.1042/BST20110658

[23] Gueneau L, Bertrand AT, Jais JP, Salih MA, Stojkovic T, et al (2009) Mutations of the FHL1 gene cause Emery-Dreifuss muscular dystrophy. Am J Hum Genet 85: 338–353. 19716112 10.1016/j.ajhg.2009.07.015 PMC2771595

[24] Liang WC, Mitsuhashi H, Keduka E, Nonaka I, Noguchi S, et al (2011) TMEM43 mutations in Emery-Dreifuss muscular dystrophy-related myopathy. Ann Neurol 69: 1005–1013. 21391237 10.1002/ana.22338

[25] Morris GE, Sewry CA, Wehnert M (2010) Molecular genetics of Emery–Dreifuss muscular dystrophy. In Encyclopedia of Life Sciences (ELS), 1–8, John Wiley and Sons, Chichester.

[26] Lam le T, Böhm SV, Roberts RG, Morris GE (2011) Nesprin-2 epsilon: a novel nesprin isoform expressed in human ovary and Ntera-2 cells. Biochem Biophys Res Commun 412: 291–295. 21820406 10.1016/j.bbrc.2011.07.085

[27] Randles KN, Lam le T, Sewry CA, Puckelwartz M, Furling D, et al (2010) Nesprins, but not sun proteins, switch isoforms at the nuclear envelope during muscle development. Dev Dyn 239: 998–1009. 20108321 10.1002/dvdy.22229 PMC3334500

[28] Bastien P, Procop GW, Reischl U (2008) Quantitative real-time PCR is not more sensitive than “conventional” PCR. J Clin Microbiol 46: 1897–1900. 18400914 10.1128/JCM.02258-07 PMC2446855

[29] Rajgor D, Mellad JA, Autore F, Zhang Q, Shanahan CM (2012) Multiple novel nesprin-1 and nesprin-2 variants act as versatile tissue-specific intracellular scaffolds. PLoS One 7: e40098. 22768332 10.1371/journal.pone.0040098 PMC3388047

[30] Morris GE, Randles KN (2010) Nesprin isoforms: are they inside or outside the nucleus? Biochem Soc Trans 38: 278–280. 20074074 10.1042/BST0380278

[31] Warren DT, Tajsic T, Mellad JA, Searles R, Zhang Q, et al (2010) Novel nuclear nesprin-2 variants tether active extracellular signal-regulated MAPK1 and MAPK2 at promyelocytic leukemia protein nuclear bodies and act to regulate smooth muscle cell proliferation. J Biol Chem 285: 1311–1320. 19861416 10.1074/jbc.M109.032557 PMC2801258

[32] Rashmi RN, Eckes B, Glöckner G, Groth M, Neumann S, et al (2012) The nuclear envelope protein Nesprin-2 has roles in cell proliferation and differentiation during wound healing. Nucleus 3: 172–186. 22198684 10.4161/nucl.19090 PMC3383573

[33] Libotte T, Zaim H, Abraham S, Padmakumar VC, Schneider M, et al (2005) Lamin A/C-dependent localization of Nesprin-2, a giant scaffolder at the nuclear envelope. Mol Biol Cell 16: 3411–3424. 15843432 10.1091/mbc.E04-11-1009 PMC1165422

[34] Zhang X, Xu R, Zhu B, Yang X, Ding X, et al (2007) Syne-1 and Syne-2 play crucial roles in myonuclear anchorage and motor neuron innervation. Development 134: 901–908. 17267447 10.1242/dev.02783

[35] Lüke Y, Zaim H, Karakesisoglou I, Jaeger VM, Sellin L, et al (2008) Nesprin-2 Giant (NUANCE) maintains nuclear envelope architecture and composition in skin. J Cell Sci 121: 1887–1898. 18477613 10.1242/jcs.019075

[36] Zhang J, Felder A, Liu Y, Guo LT, Lange S, et al (2010) Nesprin 1 is critical for nuclear positioning and anchorage. Hum Mol Genet 19: 329–341. 19864491 10.1093/hmg/ddp499 PMC2796894

[37] Lei K, Zhang X, Ding X, Guo X, Chen M, et al (2009) SUN1 and SUN2 play critical but partially redundant roles in anchoring nuclei in skeletal muscle cells in mice. Proc Natl Acad Sci USA 106: 10207–10212. 19509342 10.1073/pnas.0812037106 PMC2700906

[38] Puckelwartz MJ, Kessler E, Zhang Y, Hodzic D, Randles KN, et al (2009) Disruption of nesprin-1 produces an Emery Dreifuss muscular dystrophy-like phenotype in mice. Hum Mol Genet 18: 607–620. 19008300 10.1093/hmg/ddn386 PMC2722216

[39] Andrews PW, Damjanov I, Simon D, Banting GS, Carlin C, et al (1984) Pluripotent embryonal carcinoma clones derived from the human teratocarcinoma cell line Tera-2. Differentiation in vivo and in vitro. Lab Invest 50: 147–162. 6694356

[40] Manilal S, Sewry CA, Man N, Muntoni F, Morris GE (1997) Diagnosis of X-linked Emery-Dreifuss muscular dystrophy by protein analysis of leucocytes and skin with monoclonal antibodies. Neuromuscul Disord 7: 63–66. 9132142 10.1016/s0960-8966(96)00405-1

[41] Gey GO, Coffman WD, Kubicek MT (1952) Tissue culture studies of the proliferative capacity of cervical carcinoma and normal epithelium. Cancer Res 12: 264–265.

[42] Holt I, Nguyen TM, Wehnert M, Morris GE (2006) Lamin A/C assembly defects in Emery-Dreifuss muscular dystrophy can be regulated by culture medium composition. Neuromuscul Disord 16: 368–373. 16697197 10.1016/j.nmd.2006.03.014

[43] Shanahan CM, Weissberg PL, Metcalfe JC (1993) Isolation of gene markers of differentiated and proliferating vascular smooth muscle cells. Circ Res 73: 193–204. 8508530 10.1161/01.res.73.1.193

[44] Thomson JA, Itskovitz-Eldor J, Shapiro SS, Waknitz MA, Swiergiel JJ, et al (1998) Embryonic stem cell lines derived from human blastocysts. Science 282: 1145–1147. 9804556 10.1126/science.282.5391.1145

[45] Livak KJ, Schmittgen TD (2001) Analysis of relative gene expression data using real-time quantitative PCR and the 2(-Delta Delta C(T)) Method. Methods 25: 402–408. 11846609 10.1006/meth.2001.1262

[46] Schmittgen TD, Livak KJ (2008) Analyzing real-time PCR data by the comparative C(T) method. Nat Protoc 3: 1101–1108. 18546601 10.1038/nprot.2008.73

[47] Nguyen TM, Morris GE (2010) A rapid method for generating large numbers of high-affinity monoclonal antibodies from a single mouse. In: Walker JM, editor. The Protein Protocols Handbook, 3rd Edition. Totowa NJ: Humana Press. 1961–1974.

[48] Pereboev A, Morris GE (1996) Reiterative screening of phage-display peptide libraries with antibodies. Methods Mol Biol 66: 195–206. 8959716 10.1385/0-89603-375-9:195

