# Supplementary material for: Nesprins: Tissue-Specific Expression of Epsilon and Other Short Isoforms
Source: PLoS One. 2014 Apr 9;9(4):e94380. doi: 10.1371/journal.pone.0094380 (PMC3981789; doi:10.1371/journal.pone.0094380)
Supplement: File S1 — Contains Table S1, Quantitative PCR primer pairs. Table S2, PCR primer pairs used to span the 69 bp DV23 regions of nesprin-1 and nesprin-2 and to amplify from within the KASH region of nesprin-2. Table S3, Relative expression (RE) of nesprin-1 (N1) isoforms in human tissues and cultured cells. Table S4, Relative expression (RE) of nesprin-2 (N2) isoforms in human tissues and cultured cells. (PDF) [file pone.0094380.s001.pdf]

Supporting Information

Duong et al. "Nesprins: tissue-specific expression of epsilon and other short isoforms"

Table S1: Quantitative PCR primer pairs

Target (Accession)[a]	Primer Sequence 5' to 3'	Product Size (sequence confirmed)[b]	Efficiency (%)
**Beta-actin** (NM_001101)	F: CCCTGGAGAAGAGCTACGAG<br>R: TGAAGGTAGTTTCGTGGATGC	135bp (skeletal muscle)	100
**GAPDH** (M33197)	F: CAGCCTCAAGATCATCAGCA<br>R: GTCTTCTGGGTGGCAGTGAT	135bp (skeletal muscle)	96
**N1-giant** (NM_182961)	F: AAGCTGAAAGCGAAGACAGA<br>R: GAGCCGATTCTCAGCTTGTC	219bp (heart)	97
**N1-beta-1** (AY061755)	F: GTTCTTTCATTTTGGTGACCATC<br>R: AAGACTTTAGCTTTTTCAATCCAC	283bp (skeletal muscle, spleen)	90
**N1-beta-2** (AY184206)	F: ACCTGCCCGTCAATGTCATG<br>R: GTCGCAACTCCTCAGTCTCC	183bp (thyroid)	95
**N1-alpha-1** (AY061756)	F: CCTGATAATTTTGGCTTCAT<br>R: GTCCTCAGGCTGCTCATGTTC	268bp (spleen)[c]	ND[d]
**N1-alpha-2** (AY184203)	F: CTCCCAGATTGTAACTGCGATGT<br>R: CTATTGGCTTGGCCAGCTCT	145bp (heart)	108
**N2-giant** (NM_015180)	F: CTCTTCCAGAGCTTCACGAGG<br>R: CCATCTGCACCAGCCAGGCAC	303bp (heart, [32])	92
**N2-gamma** (AY061759)	F: AAACTTTTCCCCCAGTCTCG<br>R: TGCTTTTCTGCTGCAACATC	315bp (skeletal muscle)	95
**N2-epsilon-1** (CD654909)	F: CAGCCGCTTGAGTTGAAGG<br>R: CCATCTGCACCAGCCAGGCAC	303bp (Ntera-2, [32])	90
**N2-epsilon-2** (DB089560)	F: GGGAACTTGGAGCTTTCGCT<br>R: CGCTGTAGATCCTGCTGCTC	226bp (ovary)	97
**N2-beta-1** (AY061757)	F: AACAATTTACCCTGAGTGGATCT<br>R: CTTGAGCCAGTCCTCAAAGC	276bp (skeletal muscle)[c]	ND[d]
**N2-beta-2** (AY184205)	F: GTCTCCCCCACTAAACCGTG<br>R: GCGCTGGTTGCCCTCGTGGA	386bp (kidney)	91
**N2-alpha-1** (AY061758)	F: CTAACAACTGGATACTGTGGT<br>R: CCATCCGAAATGGATTTACC	390bp (skeletal muscle)	89
**N2-alpha-2** (AY184204)	F: CTTGCTGTCTTTCGTTTCAG<br>R: ACCTGACTGCTGCTCTGTGA	277bp (kidney)	105

Primer pairs for the endogenous controls and for different isoforms of nesprin-1 (N1) and nesprin-2 (N2).

[a] GenBank accession numbers for human sequences.

[b] Primer pairs were used in conventional PCR with the source of target cDNA as shown. Fidelity of the PCR products was confirmed by sequencing. Sequences from the nesprin-2-giant and nesprin-2-epsilon-1 primer pairs had additionally been confirmed in an earlier study [32].

[c] Conventional PCR with nesprin-1-alpha-1 and nesprin-2-beta-1 primer pairs, with the source of cDNA as shown, gave amplification of the correct products. Quantitative PCR with the same primer pairs was borderline detectable, or not detectable. For these two isoforms, several other combinations of primers were tried, which failed to give products with qPCR (see text).

[d] It was not possible to determine the efficiency of amplification of nesprin-1-alpha-1 or nesprin-2-beta-1. ND = not detected.

Table S2: PCR primer pairs used to span the 69bp DV23 regions of nesprin-1 and nesprin-2 and to amplify from within the KASH region of nesprin-2

		Product size (sequence confirmed)	
**Target**	**Primer Sequence 5' to 3'**	**"With" DV23**	**"Without" DV23**
Nesprin-1 DV23	F: CTAGATGAGCTCCGACGGTAC<br>R: CCCAGCAGTTTCATGTAGCCT	638bp<br>(skeletal muscle, vsmc)	569bp<br>(Ntera-2)
Nesprin-2 DV23<br>(All N2 isoforms except N2-alpha-1)	F: GGAGCAGCTGATTCAGAAGAG<br>R: GGTTTATAAGGGGTGCTGGA	615bp<br>(skeletal muscle)	546bp<br>(liver)
Nesprin-2 DV23<br>(N2-alpha-1 only)	F: GCCTATGAGTTGATTAATTC<br>R: CCTGTCACCTTCCATTTGCT	479bp<br>(skeletal muscle)	410bp
		**Nesprin-2 "with" KASH**	
Nesprin-2 +KASH	F: CGAAGAAGACTACAGCTGCACTC<br>R: TAGGGTGGAATGTCCTCGCTTGC	490bp<br>(skeletal muscle, fibroblasts)	

Following conventional PCR, with the source of target cDNA as shown, fidelity of the PCR products was confirmed by sequencing.

Table S3: Relative expression (RE) of nesprin-1 (N1) isoforms in human tissues and cultured cells.

Sample	N1-Giant	N1-beta-1	N1-beta-2	N1-alpha-1	N1-alpha-2
1 Adipose	134 ± 58 (3)	42 ± 8 (3)	6 ± 2 (3)	nd (2)	2 ± 0 (2)
2 Bladder	88 ± 20 (3)	6 ± 1 (3)	3 ± 0 (3)	nd (2)	<1 (2)
3 Brain	166 ± 76 (3)	1 ± 0 (3)	3 ± 1 (3)	nd (2)	<1 (2)
4 Cervix	170 ± 35 (3)	3 ± 1 (3)	3 ± 1 (3)	nd (2)	<1 (2)
5 Colon	130 ± 58 (3)	6 ± 2 (4)	6 ± 0 (3)	nd (2)	1 ± 1 (3)
6 Esophagus	46 ± 28 (3)	5 ± 1 (4)	1 ± 0 (3)	nd (2)	1 ± 1 (3)
7 Heart	148 ± 24 (3)	21 ± 9 (4)	4 ± 2 (3)	nd (2)	76 ± 19 (3)
8 Kidney	226 ± 56 (3)	7 ± 1 (3)	9 ± 5 (3)	nd (2)	1 ± 0 (2)
9 Liver	16 ± 5 (3)	1 ± 1 (3)	1 ± 0 (3)	nd (2)	1 ± 0 (2)
10 Lung	214 ± 51 (3)	12 ± 3 (3)	5 ± 1 (3)	nd (2)	2 ± 2 (2)
11 Ovary	338 ± 107 (4)	3 ± 3 (3)	4 ± 1 (3)	nd (2)	<1 (2)
12 Placenta	38 ± 11 (3)	2 ± 0 (3)	2 ± 0 (3)	nd (2)	1 ± 0 (2)
13 Prostate	206 ± 79 (4)	2 ± 1 (4)	8 ± 1 (3)	nd (2)	<1 (2)
14 Skeletal Muscle	87 ± 26 (3)	10 ± 3 (3)	1 ± 0 (3)	nd (2)	55 ± 18 (3)
15 Small Intestine	54 ± 11 (3)	3 ± 1 (3)	1 ± 0 (3)	nd (2)	<1 (2)
16 Spleen	118 ± 26 (3)	223 ± 22 (4)	12 ± 4 (3)	nd (2)	1 ± 1 (2)
17 Testis	149 ± 25 (3)	3 ± 1 (3)	12 ± 15 (3)	nd (2)	<1 (2)
18 Thymus	36 ± 6 (3)	<1 (3)	1 ± 1 (3)	nd (2)	<1 (2)
19 Thyroid	562 ± 184 (3)	16 ± 3 (3)	25 ± 9 (3)	nd (2)	<1 (2)
20 Trachea	109 ± 40 (3)	4 ± 3 (3)	3 ± 1 (3)	nd (2)	1 ± 0 (2)
21 ESC	<1 (3)	<1 (2)	<1 (2)	nd (2)	nd (2)
22 Fibroblast	19 ± 2 (3)	<1 (2)	<1 (2)	nd (2)	<1 (2)
23 LCL	3 ± 1 (3)	<1 (3)	<1 (3)	nd (2)	<1 (2)
24 Ntera-2	<1 (3)	nd (3)	<1 (3)	nd (2)	<1 (2)
25 HeLa	<1 (3)	<1 (4)	<1 (3)	nd (2)	<1 (2)
26 U2OS	11 ± 4 (3)	<1 (3)	<1 (3)	nd (2)	nd (2)
27 VSMC	166 ± 38 (3)	3 ± 0 (3)	9 ± 1 (3)	nd (2)	<1 (2)

Results of qPCR are shown as Mean Relative Expression ± SD (number of measurements). nd = Not detected.

Table S4: Relative expression (RE) of nesprin-2 (N2) isoforms in human tissues and cultured cells.

Sample	N2-Giant	N2-gamma	N2-epsilon-1	N2-epsilon-2	N2-beta-1	N2-beta-2	N2-alpha-1	N2-alpha-2
1 Adipose	340 ± 57 (3)	3 ± 2 (4)	2 ± 1 (2)	3 ± 0 (3)	<1 (3)	3 ± 1 (3)	1 ± 2 (3)	<1 (2)
2 Bladder	94 ± 35 (3)	1 ± 0 (2)	<1 (2)	3 ± 0 (3)	<1 (2)	4 ± 0 (2)	<1 (3)	1 ± 0 (2)
3 Brain	17 ± 10 (3)	<1 (3)	<1 (2)	19 ± 1 (3)	<1 (3)	<1 (2)	<1 (3)	<1 (3)
4 Cervix	251 ± 13 (3)	2 ± 0 (3)	1 ± 0 (2)	2 ± 0 (3)	<1 (5)	4 ± 0 (2)	<1 (3)	1 ± 0 (2)
5 Colon	192 ± 77 (3)	1 ± 0 (2)	1 ± 0 (2)	3 ± 0 (3)	<1 (2)	3 ± 1 (3)	<1 (3)	2 ± 1 (5)
6 Esophagus	260 ± 109 (3)	1 ± 0 (2)	2 ± 3 (2)	nd (3)	<1 (2)	2 ± 1 (3)	3 ± 2 (3)	1 ± 0 (2)
7 Heart	196 ± 77 (3)	1 ± 0 (3)	<1 (6)	42 ± 5 (5)	<1 (5)	<1 (2)	55 ± 72 (5)	4 ± 4 (3)
8 Kidney	1231 ± 182 (3)	3 ± 0 (3)	5 ± 0 (3)	3 ± 1 (3)	<1 (3)	9 ± 1 (3)	<1 (3)	36 ± 14 (3)
9 Liver	552 ± 76 (3)	<1 (2)	2 ± 1 (2)	2 ± 1 (3)	<1 (2)	8 ± 1 (3)	1 ± 1 (3)	8 ± 3 (3)
10 Lung	269 ± 43 (3)	2 ± 1 (2)	1 ± 1 (2)	9 ± 0 (3)	<1 (4)	1 ± 1 (3)	<1 (3)	3 ± 0 (3)
11 Ovary	355 ± 93 (3)	1 ± 1 (3)	34 ± 9 (6)	30 ± 4 (5)	<1 (4)	<1 (2)	1 ± 0 (3)	3 ± 3 (3)
12 Placenta	551 ± 113 (3)	3 ± 2 (3)	4 ± 2 (2)	1 ± 0 (3)	<1 (3)	9 ± 3 (3)	<1 (3)	8 ± 4 (3)
13 Prostate	212 ± 11 (3)	2 ± 0 (2)	2 ± 1 (2)	20 ± 3 (3)	<1 (4)	3 ± 2 (3)	2 ± 0 (3)	3 ± 1 (3)
14 Skeletal Muscle	636 ± 233 (3)	1 ± 0 (3)	1 ± 0 (2)	2 ± 0 (3)	<1 (3)	1 ± 0 (2)	315 ± 177 (3)	<1 (3)
15 Small Intestine	234 ± 10 (3)	2 ± 0 (2)	1 ± 0 (2)	3 ± 0 (3)	<1 (2)	1 ± 0 (2)	<1 (3)	6 ± 2 (4)
16 Spleen	197 ± 56 (3)	1 ± 0 (2)	1 ± 0 (2)	7 ± 0 (3)	<1 (3)	2 ± 1 (3)	<1 (3)	2 ± 1 (3)
17 Testis	206 ± 90 (3)	2 ± 2 (3)	12 ± 8 (3)	48 ± 1 (3)	<1 (2)	4 ± 0 (3)	1 ± 1 (3)	1 ± 0 (3)
18 Thymus	111 ± 14 (3)	<1 (2)	<1 (2)	17 ± 1 (3)	<1 (2)	1 ± 0 (2)	<1 (3)	<1 (2)
19 Thyroid	1139 ± 290 (3)	5 ± 1 (4)	5 ± 1 (6)	44 ± 10 (5)	<1 (4)	6 ± 6 (3)	9 ± 3 (3)	9 ± 5 (3)
20 Trachea	318 ± 22 (3)	2 ± 0 (2)	1 ± 0 (2)	2 ± 1 (3)	<1 (4)	6 ± 4 (3)	1 ± 1 (3)	2 ± 1 (3)
21 ESC	88 ± 44 (3)	<1 (4)	23 ± 7 (5)	<1 (3)	<1 (3)	<1 (2)	nd (3)	2 ± 0 (3)
22 Fibroblast	60 ± 12 (3)	<1 (4)	<1 (2)	<1 (2)	<1 (3)	<1 (2)	nd (3)	1 ± 0 (3)
23 LCL	193 ± 41 (3)	<1 (3)	<1 (2)	<1 (2)	<1 (3)	1 ± 0 (2)	<1 (3)	2 ± 1 (3)
24 Ntera-2	53 ± 9 (3)	<1 (4)	71 ± 10 (5)	<1 (5)	<1 (3)	<1 (2)	<1 (3)	6 ± 2 (3)
25 HeLa	99 ± 27 (4)	<1 (2)	<1 (2)	nd (2)	<1 (3)	<1 (3)	<1 (2)	3 ± 1 (3)
26 U2OS	19 ± 3 (3)	<1 (2)	<1 (2)	nd (2)	<1 (2)	<1 (2)	nd (2)	<1 (2)
27 VSMC	3 ± 1 (3)	<1 (2)	<1 (2)	nd (2)	<1 (2)	<1 (2)	nd (2)	nd (2)

Results of qPCR are shown as Mean Relative Expression ± SD (number of measurements). nd = Not detected.
